# Cardiac cellular diversity and functionality in cardiac repair by single-cell transcriptomics

**DOI:** 10.3389/fcvm.2023.1237208

**Published:** 2023-10-18

**Authors:** Wei Chen, Chuling Li, Yijin Chen, Jianping Bin, Yanmei Chen

**Affiliations:** ^1^ Department of Cardiology, State Key Laboratory of Organ Failure Research, Nanfang Hospital, Southern Medical University, Guangzhou, China; ^2^ Guangdong Provincial Key Laboratory of Cardiac Function and Microcirculation, Guangzhou, China; ^3^ Department of Cardiology, Ganzhou People’s Hospital, Ganzhou, China

**Keywords:** myocardial infarction, left ventricular remodeling, cardiac repair, single-cell transcriptomics, therapeutic approach

## Abstract

Cardiac repair after myocardial infarction (MI) is orchestrated by multiple intrinsic mechanisms in the heart. Identifying cardiac cell heterogeneity and its effect on processes that mediate the ischemic myocardium repair may be key to developing novel therapeutics for preventing heart failure. With the rapid advancement of single-cell transcriptomics, recent studies have uncovered novel cardiac cell populations, dynamics of cell type composition, and molecular signatures of MI-associated cells at the single-cell level. In this review, we summarized the main findings during cardiac repair by applying single-cell transcriptomics, including endogenous myocardial regeneration, myocardial fibrosis, angiogenesis, and the immune microenvironment. Finally, we also discussed the integrative analysis of spatial multi-omics transcriptomics and single-cell transcriptomics. This review provided a basis for future studies to further advance the mechanism and development of therapeutic approaches for cardiac repair.

## 1. Introduction

Myocardial infarction (MI) is one of the major causes of morbidity and mortality among all cardiovascular diseases (1). After a heart attack, the mammal hearts has a limited regeneration capacity to repair itself, and cardiac muscle loss is replaced by fibrotic scar tissue, followed by reverse cardiac remodeling, eventually leading to impaired cardiac function (2). Previous studies have addressed the importance of different cell subsets in cardiac repair. Comprehensive and further exploration of these cell type-specific assessments of genetic and molecular mechanisms in the heart might prompt new and precise integrated therapeutic approaches for cardiac repair (3, 4). Recently, single-cell transcriptomics has enabled us to study the expression profiles of individual cells, showing complete information on cellular heterogeneity and dynamic changes during heart development and cardiac disease. Applying this technology to cardiac development and repair might lead to discovering new cell subtypes, molecular changes, and therapeutic targets relevant to cardiac repair (5). So far, a serial of reviews had summarized the cardiovascular development, cardiac stem/progenitor cell, numerous heart diseases including MI, cardiomyopathy, congenital heart defect, and so on (6–8). However, there is still a lack of comprehensive review on recent advances in cardiac repair via applying single-cell transcriptomics (9).

In this review, we focus on recent advances in cardiac repair via applying single-cell transcriptomics and new assistive tools and technology upgrades for single-cell transcriptomics, especially spatial single-cell omics. This review summarized the cardiac cellular landscape and the related molecular mechanisms involved in cardiac repair at single-cell level, which will help to facilitate the emergence of new therapies that could promote cardiac repair.

## 2. Insights on cardiac regeneration in cardiac repair by single-cell transcriptomics

Triggering cardiac regeneration represented as one of most promising regeneration strategies for cardiac repair. There are two most widely therapeutic approaches to achieve the cardiac regeneration: promotion of preexisting cardiomyocyte proliferation and *in situ* reprogramming of fibroblasts to cardiomyocytes (10). We reviewed recent advancements in these two approaches by applying single-cell transcriptomics.

### 2.1. Promotion of preexisting cardiomyocyte proliferation

Cardiomyocytes are the most abundant in the heart as compared to fibroblasts, endothelial cells and immune cells. Emerging evidence revealed that regenerating cardiomyocytes are mainly from preexisting cardiomyocytes instead of resident stem cells in injured hearts (11). Significant genetic and functional heterogeneity exist among preexisting cardiomyocytes; therefore, exploring the contributions of cardiomyocyte subtypes to the underlying regenerative processes at single cell resolution is necessary (12, 13). Hu et al. applied sNucDrop-seq (highly scalable single-nucleus RNA-seq) to investigate the transcriptome changes of postnatal hearts in mice (Table 2). They divided cardiomyocytes into two groups, including less mature type or developing type (dCMs) with immature cardiomyocyte markers such as Myocd (also known as myocardin) and more mature type (mCMs) with abundant mitochondria and positive for muscle fiber markers such as Actc1 (also known as cardiac α-actin) (Table 1) (14). Interestingly, some mCMs, such as those in the mCM1, also expressed these fibroblast-enriched markers (e.g., Col1a2, Col3a1, and Dcn), and Gata4^+^ or Myocd^+^ nuclei were significantly enriched only in dCMs but not in mCMs or nonmyocyte cells.Partially different from the widely established markers of cardiomyocyte proliferation such as nucleotides analogs (EdU, BrdU), M-phase markers AURKB (Aurora B kinase) and pHH3 (phospho-histone H3), this study also entified a small population of presumably proliferating cardiomyocytes (pCMs) that expressed cell cycle genes (e.g., Mki67, Cenpp, and Kif15) (Table 1), and found both P6 and P10 pCMs populations have the same similar gene signatures of cell proliferation: mitosis, G2/M transition, chromosome segregation and cytokinesis (14–16). A similar study explored whether a unique subset of preexisting cardiomyocytes could be regenerated by applying snRNA-seq (single-nucleus RNA sequencing) in healthy, injured and regenerating mice hearts (17) (Table 2). Five major clusters (CM1–CM5) were identified as cardiomyocytes based on the established marker of Myh6 (Table 1). Among them, the immature CM4 cells express higher levels of markers of immature hearts, including Tnni1, Myh7, and Actc1, which is mainly expressed in regenerative hearts. In addition, cell-cycle genes, such as Mki67 and Ccnb1 (Table 1), were strongly activated in CM4 cells at 3 days post-MI, suggesting that CM4 may be the only regenerative subset. To further identify the upstream regulators of CM4, they performed scATAC-seq (single-cell ATAC sequencing) and identified NFYa and SRF as regulators of cardiomyocyte proliferation, and NFE2L1 and NFE2L2 as regulators of cardiomyocyte survival (13) (Table 2).

**Table 1:** List of established markers and newly identified markers in the review.

Cell type	Established markers	New identified markers
Mature cardiomyocyte	cTnT, Myh6, Ryr2, Cacna1c, TTN, KCNJ2, and ATP2A2	Myl2, Myl3, Fabp3, Fabp3, Fapb4, Uqcr11, Cox6c, Tgfbi, Igfbp3, Isg15, and Adm, Col1a2, Col3a1, and Dcn
Immature or developing cardiomyocyte	Tnni1, Myh7, and Actc1	Gata4, Myocd
Proliferative cardiomyocyte	EdU, BrdU, Aurora B, pH3, Ki-67	Kif15, Cenpp, Mki67 and Ccnb1
Fibroblast	DDR2, ACTA2, Vimentin, Periostin (POSTN), Col1a1, Col1a2, FAP, Fsp-1	Cilp, Thbs4, CTHRC1, CILP1
Endothelial cell	VWF, CD31	NPR3, FABP4, Slit2, CD41, CD157, Pdgfb
Macrophage	CD45, CD68, F4/80 M2: CD206, CD163 M1: CD80, CD86	CD72, Ms4a7, Fcrls
Neutrophil	CD45, CD66b	Icam1, Siglecf, Ifitm2, Pglyrp1, Slpi
B cell	CD45, CD19, CD20	Cd69, Ccr7, Cxcr5
DC cell	CD45, CD83	Fscn1, Ccr7

**Table 2:** Main characteristics of technologies described in the review.

Technology	Data types provided	Characteristics
snRNA-seq	mRNA	detect mRNA of the nucleus, allows transcriptomic profiling of frozen tissue
scRNA-seq	mRNA	detect the complete number of intact RNA, suitable for immune cells
sNucDrop-seq	mRNA	a droplet microfluidics-based massively parallel snRNA-seq method, free of enzymatic dissociation and nucleus sorting
spatial single-cell omics	mRNA, protein	spatiotemporal molecular and dynamic transcriptomic changes
seq-FISH	mRNA, protein	different fluorescent probes to characterize spatial organization of cells
scATAC-seq	chromatin	tag and fragment DNA sequences in open chromatin regions with DNA transposase (Tn5)
scDNase-seq	chromatin	DNase I that digest chromatin fragmentation
scMNase-seq	chromatin	MNase for detecting chromatin accessibility and nucleosome position
iscDNase-seq	DNA	barcoding DNA ends with TdT terminal transferase and T4 DNA ligase combined
CITE-seq	mRNA, protein	antibody conjugates bound to biotinylated DNA barcodes

**Figure 1:**
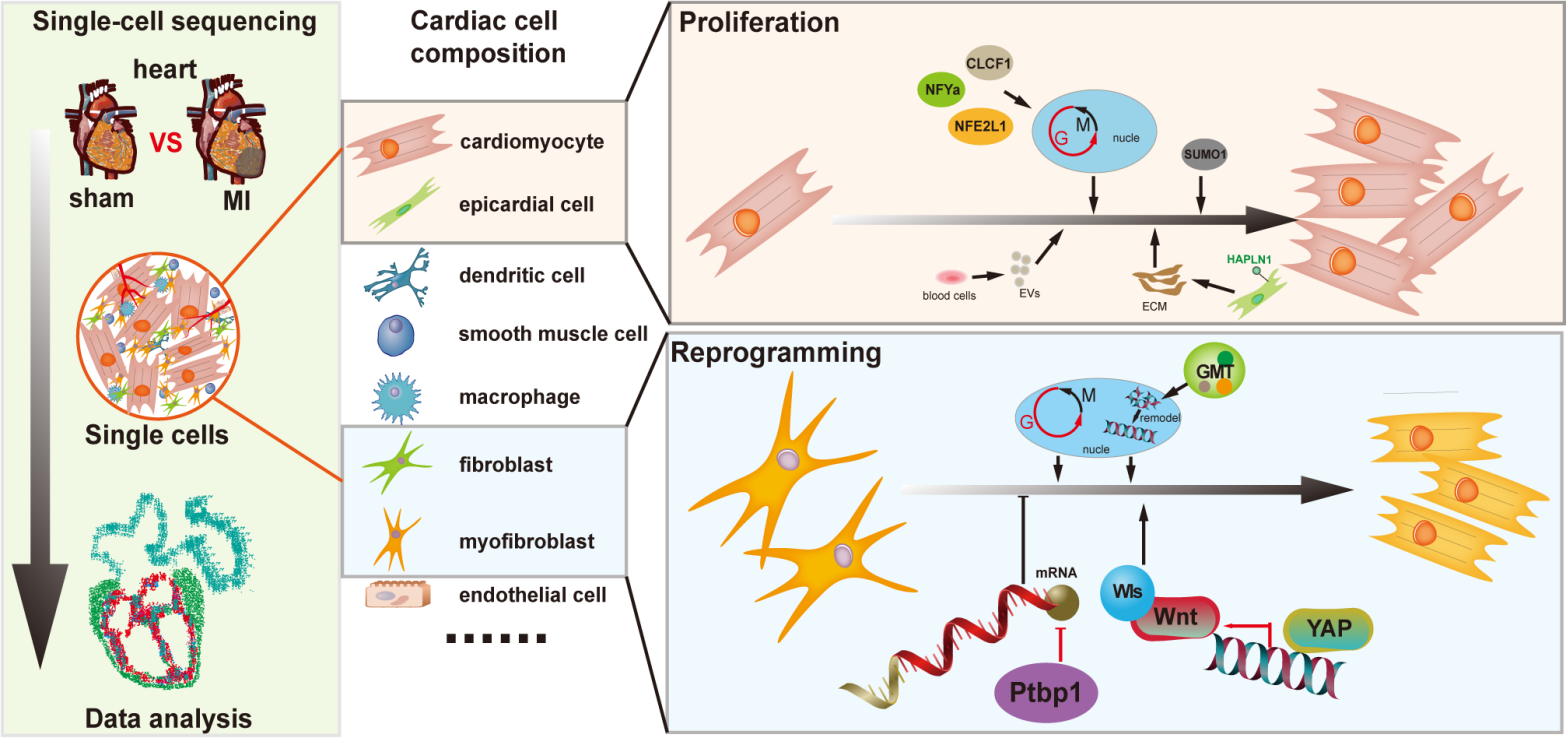
Insights on cardiac regeneration in cardiac repair by single-cell transcriptomics.

Thus, the above studies clearly reinforce the small subset of cardiomyocytes exerting proliferative capacity after injury (Figure 1). The internal increase in these cardiomyocyte subsets following injury might stimulate new strategies for cardiac regeneration.

### 2.2. Cardiac reprogramming in cardiac regeneration

Direct cardiac reprogramming offers a novel therapeutic option to regenerate injured hearts by directly converting endogenous cardiac fibroblasts into CM-like cells.

However, the underlying molecular mechanisms of this reprogramming process remains largely elusive. Characterizing inherent heterogeneity and asynchronous nature of the reprogramming process might help study the reprogramming process. Qian et al. used single-cell RNA sequencing to analyze global transcriptome changes at early stages during the reprogramming of mouse fibroblasts into induced cardiomyocytes (iCMs) and multifarious cell subsets were found to be unexpectedly downregulated in mRNA processing and splicing factors (18). Detailed functional analysis of the splicing factor in this process, Ptbp1 was proven to be a barrier for fibroblasts acquiring cardiomyocyte-specific splicing patterns. Concerning the mechanism behind this phenomenon, more single-cell data uncovered that, through Wnt signaling, YAP can participate in the communication between cardiomyocytes and fibroblasts, thus, promoting heart regeneration (19). Integrating single-cell transcriptomic of mice hearts at multiple postnatal stages, fibroblasts were identified as key constituents promoting cardiomyocyte maturation in the microenvironment (20) (Figure 1).

Another major challenge in cardiac reprogramming is the low cardiomyocyte induction efficiency. Ectopic expression of cardiac reprogramming factor combinations may improve reprogramming efficiency. In addition, single-cell assays could help identify cell heterogeneity and response to reprogramming factor combinations, which might explore the mechanisms that facilitate reprogramming effectively. Srivastava et al. performed scRNA-seq on Thy1-positive cells to determine the molecular mechanisms of reprogramming factors: GMT (Gata4(GATA binding protein 4), Mef2C (myocyte enhancer factor 2C), and Tbx5(T-box transcription factor 5)), which induce the conversion of cardiac fibroblasts into cardiomyocytes. This study demonstrates that this technology help identify the individual endpoint and determine the molecular resolutions of these cellular trajectories (21) (Figure 1).

Further investigations to determine the regulatory factors or mechanisms of this process will aid in promoting reprogramming targeting properties and efficiency for cardiac repair.

## 3. Insights on myocardial fibrosis in cardiac repair by single-cell transcriptomics

Myocardial fibrosis is a common pathophysiologic companion of MI which replaced the dead CMs and stretched the cardiac interstitium through deposition of extracellular matrix proteins (22). Currently, the subpopulations with distinguishing molecular features, cellular functions, and intercellular interactions of fibrosis in cardiac repair remain largely unexplored (23). Single-cell transcriptomics analysis of CFs provides a blueprint for interrogating the molecular and cellular basis of fibrosis in cardiac repair.

### 3.1. New subset of fibroblasts in myocardial fibrosis during cardiac repair

Among this process, fibroblasts proliferation increased and was involved in excessive scarring and contractile dysfunction, indicating that fibroblasts play a key role in myocardial fibrosis. However, the specific subpopulations of fibroblasts that drive cardiac fibrosis and the relative function of different fibroblast subpopulations on cardiac fibrosis remained unclear.

Conventionally, the cell population of smooth muscle actin–expressing (ACTA2^+^) myofibroblasts is recognized as a key mediator of early cardiac fibrosis (Table 1) (24). Recently, Pinto, et al. identified 2 previously undescribed cardiac fibroblast populations that are key drivers of fibrosis, Fibroblast-Cilp and Fibroblast-Thbs4, which emerged after induction of tissue stress to promote fibrosis. These two sub-populations represent a cell state arising primarily from resident fibroblasts rather than infiltration or proliferation of cells. Another similar study also characterized the subpopulations of human cardiac fibroblasts and revealed a previously uncharacterized population of fibroblasts that lacked the canonical markers of fibroblast activation, POSTN or fibroblast activating protein (FAP) (Table 1). Even though these two subsets were widely distributed in the heart, their lacking did not show overt signs of clinical dysfunction. Ruiz-Villalba's team also define CF heterogeneity and its role in the fibrotic healing response after MI and identify a new subpopulation of CFs expressing high levels of CTHRC1 (a collagen triple helical repeat containing 1) that emerges after MI in mice by using scRNA-seq (25). This new CTHRC1^+^ CF subpopulation were found to be essential during cardiac repair and were conservative in swine and humans, suggesting that regulation of this subpopulation might be a potential target in patients with MI. Experiments of mouse identified IL-11-positive fibroblasts subsets that play an essential role in fibroblast differentiation (26, 27). Analysis of three single-cell transcriptomics data revealed that CILP1 (cartilage intermediate layer protein 1) was specifically secreted from fibroblasts to promote myocardial fibrosis via the mTORC1 (mechanistic target of rapamycin complex 1) pathway in heart (28). Another study also applied scRNA-seq in healthy and injured adult mouse hearts which identified two previously unknown fibroblast populations termed fibroblast-Wnt expressing (F-WntX) and fibroblast-transitory (F-Trans) (29). These novel myofibroblast subtypes expressed pro-fibrotic or anti-fibrotic signatures, suggesting potential molecular or cellular targets for promoting cardiac repair.

**Figure 2:**
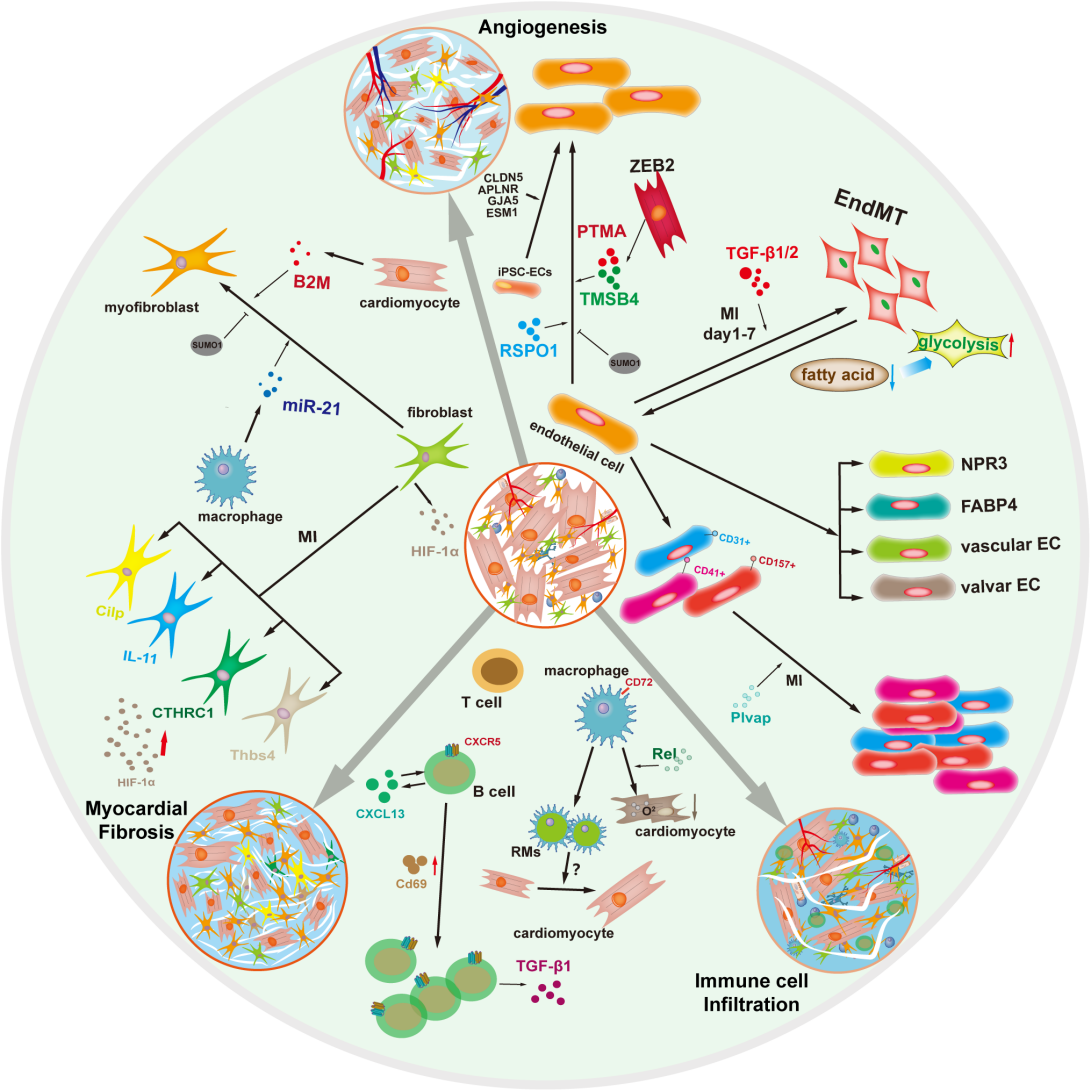
Myocardial fibrosis, angiogenesis, and immune microenvironment in cardiac repair by single-cell transcriptomics.

Currently, although many newly defined cell subsets are discovered by applying single-cell transcriptomics, these cell subsets are not be thoroughly validated in these studies. Overall, the cardiac fibroblast subpopulation varies in composition and function (Figure 2) and targeting these specific subpopulations might reverse adverse ventricular remodeling and heart failure.

### 3.2. The role of interstitials in myocardial fibrosis

Cardiac interstitial cells, including immune cells, endothelial cells, smooth muscle cells, fibroblasts and others, provide significant regulations in cardiac repair (29). To define the compendium of these cell and the response to cardiac injury, Furtado et al. applied unbiased scRNA-seq of interstitial cells from infarcted hearts and revealed 16 main subclusters. To further reveal the population dynamics in cardiac repair, they detected the change from the early injury response to myofibroblasts as a critical determinant of reparative outcome: cardiac rupture or pathological remodeling. They found potential targets for anti-fibrotic treatment and helped identify the features with susceptibility and resilience to cardiac rupture (30). The above studies suggest the indispensable function of interstitial cells in in myocardial fibrosis (Figure 2).

## 4. Angiogenesis in cardiac repair by single-cell transcriptomics

After MI, it is of great significance to promote angiogenesis to restore the vascular network for endogenous myocardial regeneration and repair of myocardial fibrosis (31). We will summarize the advance on angiogenesis in cardiac repair by single-cell transcriptomics.

### 4.1. Angiogenesis related cell types

Angiogenesis and vascular regression precede myocardial fibrosis (32). It is essential to promote angiogenesis to restore the vascular network for endogenous myocardial regeneration and myocardial fibrosis repair (33). Cardiac endothelial cells are abundant in the heart and essential for angiogenesis, and VWF and CD31 are the common markers of endothelial cells (Table 1) (34, 35). Using single-cell transcriptomics, ECs had cellular and molecular heterogeneity and were identified as four subtypes, including NPR3^+^ (natriuretic peptide receptor 3) ECs, with FABP4^+^ (fatty acid binding protein 4) coronary vascular ECs, vascular ECs, and valvar ECs (36). Some studies have revealed that ECs can be grouped into ten clusters by mean of distinct gene signatures. Another study analyzed single-cell transcriptomics of neonatal mice heart cells and revealed that endothelial cells can differentiate into subsets distinct from those previously studied (37). The subpopulation of endothelial cells is still abundant and worth exploring for their functions and mechanisms of angiogenesis. Wang et al. also found that endothelial cells in the neonatal heart are heterogeneous and identified six clusters of endothelial cells: Art.EC, VEC1, VEC2, VEC3, Endo, and Pro.EC. The Pro.EC expressed high levels of cycle genes included Mki67 and Cenpa (38).

iPSC-ECs (Human induced pluripotent stem cell-derived endothelial cells) have emerged as an effective therapeutic approach for angiogenesis (39). Purified iPSC-ECs were transcriptionally heterogeneous with four major populations, marked by CLDN5, APLNR, GJA5, and ESM1 using droplet-based scRNA-seq (40). In addition, the Bona iPSC-ECs exhibited endothelial morphology and function, including tube formation, response to inflammatory, and production of NO. Growth factors secreted by acidic and basic fibroblasts are involved in angiogenesis following cardiac repair. Cardiomyocytes and ECs are in close proximity and contact through the paracrine signaling and direct communication (41). Moreover, ZEB2-expressing cardiomyocytes were enriched for TMSB4 (paracrine thymosin beta 4) and PTMA (prothymosin alpha), which enhance EC migration and stimulate angiogenesis (42) (Figure 2).

### 4.2. Mechanisms and therapeutic potential of angiogenesis in cardiac repair

Currently, the molecular mechanisms and kinetics of ECs in response to ischemic injury are not well established. Tombor et al. reveal that ECs undergo transient activation with a metabolic switch from fatty acids towards glycolytic metabolism within the first days after MI. This transient activation may facilitate endothelial cells proliferation and migration to regenerate the blood vessels (43). Pdgfb is considered to be a marker of endothelial cells and closely related to angiogenesis (44). After EMT (epithelial-mesenchymal transition), Slit2^+^ EPDCs (slit guidance ligand 2^+^ epicardium-derived cell) functioned as vascular guideposts sustaining the persistence of mature angiogenic EC, which were controlled by paracrine signaling from EPDCs (45). Further elucidation of bone marrow vascular niche responses with CD31^+^ ECs revealed that ECs with high endomucin expression mediate IL-MI Post-1β-dependent pyroptosis. The loss of these cells is associated with CD41^+^ myeloid progenitor expansion and can be prevented by anti-inflammasome inhibitors (46). Additional studies have suggested that CD157^+^ ECs, acted as resident vascular endothelial stem cells, can clonally expanding in response to cardiac injury (47). Overall, single-cell transcriptomics would be necessary to find out the new subsets and secretive mutual effect of ECs for accelerating angiogenesis.

## 5. Immune microenvironment in cardiac repair

It is well known that immune cells were extensively involved in inflammation and remodeling following MI. Recent advances in single-cell transcriptomics offers us an unbiased approach to investigate the time-dependent composition, cellular response, heterogeneity, and dynamics of immune cells, providing clues for the establishment of new biomarkers and therapeutic approaches to cardiac repair.

### 5.1 Role of macrophages in cardiac repair by single-cell transcriptomics

Immune cells mediate-immune microenvironment is reported to be a double-edged sword in the cardiac repair. Macrophages, the specialized mononuclear phagocytes with the hematopoietic lineage marker CD45 (Table 1) (48), stay at the myocardium from the earliest developmental heart and are vital in response to ischemia injury (49). Increasing evidence has uncovered that single-cell transcriptomics of cardiac-RMs (self-renewing resident macrophages) proved that *in situ* proliferation and transcriptional activation of some cardiac-RMs directly correlate with increased cardiomyocyte growth. Further sub-clustering analysis separated the macrophages into M1 and M2 populations, inconsistent with previous studies (50). To explore the contributions of macrophages to regenerative neonatal hearts, the team of Olson performed scRNA-seq and on hearts at different time points post-MI of regenerative and non-regenerative mice. They revealed dynamic compositional changes of immune-related cell types during post-MI injury response. This study also integrated analysis of scRNA-seq and scATAC-seq data to uncover the underlying dynamics of open chromatin landscapes and regenerative gene regulatory networks of nonmyocyte subtypes (17).

To exploring macrophages in the neonatal myocardium, some studies have focused on their role in the adult myocardium. By applying genetic fate mapping and scRNA-seq, the healthy adult myocardium was revealed to contain four populations of macrophages (51). Resident macrophages have functions similar to those of neonatal macrophages, which could promote angiogenesis and cardiomyocyte proliferation. After MI, the number of resident macrophages in the infarct zone markedly reduced during the first few weeks post-MI. The proportion of RMs in the peri-infarct zone is 2%–5%; nonetheless, their depletion impairs cardiac function and worsens the infarct healing (52).

Macrophages play a crucial role in cardiac repair and affect heart failure progression and outcomes via different subtypes. Ni et al. identified that CD72^hi^ cardiac macrophages are the subset of pro-inflammatory macrophages associated with cardiac injury. A series of follow-up studies demonstrated that CD72^hi^ macrophages induce myocardial oxidative stress and apoptosis driven by the transcription factor: Rel (NF-kB subunit) (8).

The above studies have revealed that cardiac macrophages play an essential role in cardiac repair. These newly discovered subsets, functions, and regulatory mechanisms of macrophages are promising novel treatment avenues for cardiac repair (Figure 2).

### 5.2 Role of neutrophils, B-cell, and DC cells in cardiac repair

In addition to macrophages, other immune cells are worth noting in the context of immune responses in MI heart. Previous studies have reported neutrophils recruited into the infarcted area of the MI heart for the first time (53). Data of scRNA-seq and ST-seq (spatial-transcriptome sequencing) revealed that neutrophils were the largest cell population in the infarcted area at day 1 after MI (41.2%). However, they decreased rapidly on days 3, 5, and 7 (18.7, 10.6, and 9.5% separately). They further identified five populations and observed that clusters 2 (Icam1 and Siglecf) and 3 (Ifitm2, Pglyrp1, and Slpi) were relatively abundant on days 3 and 5 after MI surgery. Cluster 2 was indicated an important population in the post-MI immune responses. Further in-depth exploration of cluster 2 would help to discover novel therapeutic targets for cardiac repair (54).

The dendritic cells (DCs) also exhibit heterogeneity and dynamic changes in infarcted hearts. Jung et al. identified six distinct sub-clusters of DCs, three of which were predominant before MI. The sub-clusters with macrophage (Ms4a7 and Fcrls) and migratory DC (Fscn1 and Ccr7), was relatively high in the late stage. Whether both subsets of DCs are involved in injury repair post-MI requires further exploration (55).

Other immune populations, such as B and T cells, influence inflammation and remodeling following MI. A recent study performed scRNA-seq of B cells in the heart and mediastinal lymph nodes to assess the phenotyping of B-cell responses to the heart injury. A rapid accumulation of diverse B-cell subsets was observed in infarcted murine hearts at day 5 post-MI, and a heart-associated B cells, was identified exclusively in the heart. These hBs are classified by high levels of Cd69, Ccr7 (C-C-chemokine receptor type 7), Cxcr5 (CXC-chemokine receptor type 5), and TGF-β1 (transforming growth factor beta 1). In contrast, these polyclonal B cells display no sign of antigen specificity infiltrating the heart post-MI via the CXCL13-CXCR5 axis and contribute to local TGF-β1 production (56). One result demonstrated that ablation of CD4^+^ but not CD8^+^ T cells promotes heart regeneration in juvenile mice; and CD4^+^ T cells play a distinct role in the regulation of heart regeneration and repair during development (57).In contrast to this study, Jung et al. did not observe the B cells and T cells clusters in the infarcted area (58, 59).

These researches highlight that different cell subsets of immune cells have different roles in cardiac repair (Figure 2).

## 6. Cross-talk between cell types in cardiac repair

Cardiac repair involves multiple cell types and complex intercellular interactions, and single-cell transcriptomics allows detailed investigation of these multicellular microenvironments. Characterization of intercellular communication during cardiac repair is essential to understanding the cardiac development and normal organ function, so that formulating precise therapeutic tactics to promote cardiac repair. Intercellular communication between fibroblasts and cardiomyocytes is most common and vital during cardiac repair. It is mainly mediated by paracrine factors including cytokines or growth factors, direct contacts with gap junctions, or indirect interaction by mean of extracellular matrix (ECM) proteins (60). Increasing studies have consistently proved that fibroblasts are the central hubs of intercellular communication. A recent study revealed that cardiac fibroblasts were identified as key constituents promoting cardiomyocyte maturation in the microenvironment by integrating single-cell transcriptomic of mice hearts at multiple postnatal stages (20). Molenaar et al. applied scRNA-seq and used a dataset of ligand-receptor pairs to define the potential intercellular communication of cardiac cells after ischemic injury. They observed that stressed cardiomyocytes following IR (ischemia reperfusion) could secrete multiple unstudied factors to communicate with other cells. Subsequent *in vitro* experiments confirmed that cardiomyocytes secrete beta-2 microglobulin to activate fibroblasts in response to ischemic damage (61). Another study that analyzed the ligands and receptors in different cell in three scRNA-seq datasets of non-cardiomyocytes isolated from post-MI or sham surgery also demonstrated that fibroblasts are the core cells with dense connections to multiple cardiac cells by secreting predominant ligands (62).

ECs adjust to injury and how ECs communicate with cardiomyocytes contributes to repair and regeneration (63). Furthermore, ECs up-regulated expression of secreted factors,including Tgfbi, Igfbp3, Isg15, and Adm, which decreased proliferation and increased maturation in cardiomyocytes (64). ECs show open chromatin of certain cardiomyocyte signature loci, express myofibrillar genes and cardiac-specific transcription factor MEF2C (21). Co-culture of human-induced pluripotent stem cell-derived cardiomyocytes with ECs induced MYL7 and MYL4 expression as well as NOTCH and BMP signaling in endothelial cells (65).

Many studies have reported the contributions of immune cells in cardiac regeneration. Olson et al. performed scRNA-seq at different day post-MI and revealed that the macrophage-secreted factor CLCF1 in response to injury, which communicate with cardiomyocyte and promote cardiomyocyte proliferation (38). Jung et al. explored the spatiotemporal dynamics of post-MI immune cell and revealed that the macrophages subset of Trem2hi could actively scavenge cardiomyocyte-ejected dysfunctional mitochondria improve cardiac remodeling and function by the anti-inflammatory ability, and is specifically dominant in the late-stage of infarcted hearts (66).

The interactions between myocardial cells through the secretion of paracrine signals and direct cell-to-cell contact in heart (Figure 2). This balance of crosstalk can regulate the microenvironment of myocardial regeneration during the onset and progression of cardiac repair.

## 7. Limitation of single-cell transcriptomics and complementary technologies

### 7.1. Limitation of single-cell transcriptomics

Although single-cell transcriptomics provided an unprecedented level of resolution in the assessment of cell demographics during cardiac repair, However, its limitations are also obvious: (1) The separation and purification methods of different tissue samples are different, which results in different data quality; (2) This supporting technology has very high request to the precision and precision of the instrument, while the industrial development made it; (3) However, current techniques have not completely solved the challenge of potential cell-size limitations associated with single-cell transcriptomics platforms; (4) Single-cell omics is moving from two-dimensional to three-dimensional, with higher requirements and costs for analytical instruments, which is a substantial burden for laboratories and organizations. Recently, single-cell, single-nucleus and spatial transcriptomics accompanied by multi-omics analysis is likely to contribute to the identification of markers for diagnostic and therapy of cardiac repair.

### 7.2. Spatial single-cell omics technology displays a three-dimensional view of the heart

Current single-cell methods rely largely on cell isolation from tissues, thereby losing key spatial information regarding regulatory processes. Spatial transcriptome sequencing (ST) could determine spatial expression of genes. Recently, several studies have used integrative analysis of spatial transcriptomics to provide insight into spatiotemporal molecular and dynamic transcriptomic changes after MI. Kuppe et al. provided an integrated spatial multi-omics map of human cardiac remodeling according to the gene expression, chromatin accessibility, and spatial profiling. They uncovered five populations of ventricular cardiomyocytes (vCM1, “non-stressed”; vCM2, “pre-stressed”; vCM3, “stressed”; vCM4 and vCM5), five subtypes of endothelial cells, four sub-clusters of fibroblasts, and five sub-clusters of myeloid cells, which interact each other in space to coordinate their functions. A cluster of cardiomyocytes with enriched of Mki-67 was mainly recovered in the ischemic zone (54). A similar study also performed an analysis of spatial transcriptomics and snRNA-seq in murine MI heart. They classified cardiomyocytes into three clusters: Cluster 0 and 1 were cardiomyocytes under normal conditions, whereas cluster 2 was mainly in the BZ area (border zone). More importantly, they found that transcriptional responses of mechano-sensing genes in the BZ area play a preventive role against the structural remodeling (54, 66). Recent studies have applied an image-based single-cell spatial transcriptomic, namely seq-FISH (sequential fluorescence *in situ* hybridization), demonstrating spatially resolved gene expressions and spatial mechanisms by adding the cell surface phenotype with antibody sequencing (67).

Direct spatial transcriptomic techniques can monitor spatial heterogeneity, but the complexity and repeatability in these studies is still to be determined (68). With the increasing number of available samples and technological advances, the need for spatial single-cell omics will only increase, and the data may be used to improve the disease map.

### 7.3. Multi-omics analysis technical remedy the deficiencies of single-cell transcriptomics

Despite the rapid development and wide application of single-cell transcriptomics, we cannot ignore the low per-cell transcript complexity and technical defects. These technologies include single-cell chromatin accessibility, DNA methylomics, and proteomics, which carry unique information that cannot be captured fully using scRNA-seq alone, even at its maximal depth and throughput (7).

**Figure 3:**
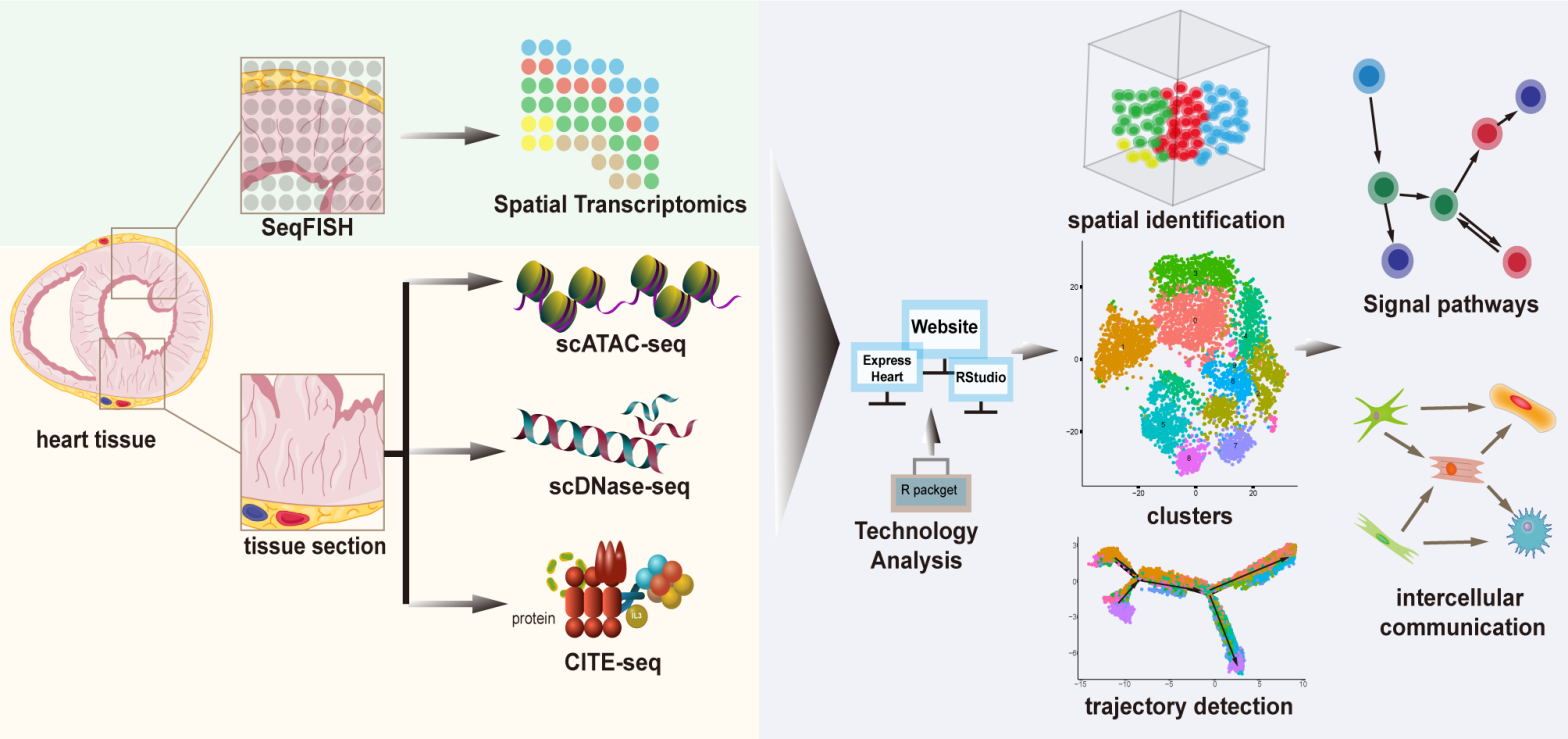
A framework of complementary technologies and analytical methods of cardiac single-cell transcriptomics.

To characterize the cellular identity at the epigenome level, studies applied scATAC-seq and miRNA-seq to compare the transcriptomic, miRNA expression, and chromatin accessibility profiles. Several techniques have been developed to detect chromatin accessibility, including scDNase-seq using DNase I that can digest chromatin fragmentation, and scMNase-seq using MNase for detecting chromatin accessibility and nucleosome position. However, owing to cell throughput of scDNase-seq is very low, the application in single-cell studies is limited. To address it, researchers designed a novel indexing strategy that avoids expensive equipment for automation or microfluidics to analyze over 15,000 cells in an experiment. This new strategy, indexing scDNase-seq (iscDNase-seq), involves barcoding DNA ends with TdT terminal transferase and T4 DNA ligase combined. Importantly, scDNase-seq data can better predict cellular heterogeneity than scATAC-seq data (69) (Figure 3; Table 2).

To characterize cellular protein levels, integrative methods allowing the simultaneous interrogation of single-cell transcriptomes along with tens of proteins have been developed using oligonucleotide-conjugated antibodies, known as CITE-seq (cellular indexing of transcriptomes and epitopes). However, these approaches are currently limited to a few surface proteins and rely on the availability of custom-designed antibodies (70) (Figure 3; Table 2).

Thus, constantly developing technologies may salvage and provide deeper and more useful information on cardiac single-cell transcriptomics. Integrating multi-omics technologies presents many challenges; nevertheless, it is expected to provide a more comprehensive picture of cellular response and activity with existing methods in the laboratory and clinic. Importantly, as single-cell analyses expand to include omics data of other types and preserve the spatiotemporal features of these data, therapeutics for cardiac repair rapidly evolve, and additional breakthroughs are anticipated in the coming years.

## 8. Conclusions and future directions

Single-cell transcriptomics provided the novel insights into the cardiac cellular landscape and dynamic molecular changes at single cell level during tissue development and disease progression, allowing more accurate tailoring of a patient's treatment. This review provided the new insight of cardiac regeneration, myocardial fibrosis, angiogenesis, and the immune microenvironment during cardiac repair revealing by use of single-cell transcriptomics. In addition, we discussed complementary technologies for analysis of cardiac single-cell transcriptomics the integrative analysis of spatial multi-omics transcriptomics and single-cell transcriptomics. This information can improve our understanding of cardiac cellular landscape and underlying pathobiological mechanisms during cardiac repair, not only guide the identification of prognostic biomarkers for cardiac injury, but also facilitate to discover novel therapeutic targets for developing innovative therapeutic strategies.

There are some challenges on the research of cardiac repair by applying single-cell transcriptomics are remained to be solved. First, current techniques have not completely solved the challenge of potential cell-size limitations associated with single-cell transcriptomics platforms; Second, there is no consistent standard for the definition of cell subsets, which may result in different markers for the same cell subset; In addition, the newly defined cell subsets are required thorough validation. Whether the newly defined cells are a previously undefined state in canonical cardiac cell activation or are instead an entirely noncanonical form of cardiac cell will be the focus of future work. Moreover, cross-talk between different cell subsets is crucial for cardiac repair. Future study should focus on how to regulating the cellular communication processes for promoting cardiac repair. Finally, but not last, the analysis methods of different studies are not consistent, which makes the integration difficult. An urgent need exists to integrate datasets and accelerate the clinical transformation research. In fact, applying these single-cell technologies in basic and translational researches is expected to grow rapidly with the continued improvement and standardization of experimental and analytical techniques. With the advance of single-cell analyses transcriptomics, spatial transcriptomics accompanied by machine learning-based analysis, therapeutics for cardiac repair will rapidly evolve, and additional breakthroughs are anticipated in the coming years.
