# Supplementary material for: Cardiac cellular diversity and functionality in cardiac repair by single-cell transcriptomics
Source: Front Cardiovasc Med. 2023 Oct 18;10:1237208. doi: 10.3389/fcvm.2023.1237208 (PMC10619858; doi:10.3389/fcvm.2023.1237208)
Supplement: Supplementary file 1 [file Datasheet1.pdf]

<table>
<tr><th>Cell type</th><th colspan="2">Established markers</th><th>New identified marker</th></tr>
<tr><td>Mature cardiomyocyte</td><td colspan="2">cTnT, Myh6, Ryr2, Cacna1c, TTN, KCNJ2, and ATP2A2</td><td>Myl2, Myl3, Fabp3, Fabp3, Fapb4, Uqcr11, Cox6c, Tgfbi, Igfbp3, Isg15, and Adm, Col1a2, Col3a1, and Dcn</td></tr>
<tr><td>Immature or developing cardiomyocyte</td><td colspan="2">Tnni1, Myh7, and Actc1</td><td>Gata4, Myocd</td></tr>
<tr><td>Proliferative cardiomyocyte</td><td colspan="2">EdU, BrdU, Aurora B, pH3, Ki-67</td><td>Kif15, Cenpp, Mki67 and Ccnb1</td></tr>
<tr><td>Fibroblast</td><td colspan="2">DDR2, ACTA2, Vimentin, Periostin (POSTN), Col1a1, Col1a2, FAP, Fsp-1</td><td>Cilp, Thbs4, CTHRC1, CILP1</td></tr>
<tr><td>Endothelial cell</td><td colspan="2">VWF, CD31</td><td>NPR3, FABP4, Slit2, CD41, CD157, Pdgfb</td></tr>
<tr><td>Macrophage</td><td rowspan="4">CD45</td><td>CD68, F4/80
M2: CD206, CD163
M1: CD80, CD86</td><td>CD72, Ms4a7, Fcrls</td></tr>
<tr><td>Neutrophil</td><td>CD66b</td><td>Icam1, Siglecf, Ifitm2, Pglyrp1, Slpi</td></tr>
<tr><td>B cell</td><td>CD19, CD20</td><td>Cd69, Ccr7, Cxcr5</td></tr>
<tr><td>DC cell</td><td>CD83</td><td>Fscn1, Ccr7</td></tr>
</table>

Table1. The list of established markers and newly identified markers in the review.

**Technology**	**Data types provided**	**Characteristics**
**snRNA-seq**	mRNA	detect mRNA of the nucleus, allows transcriptomic profiling of frozen tissue
**scRNA-seq**	mRNA	detect the complete number of intact RNA, suitable for immune cells
**sNucDrop-seq**	mRNA	a droplet microfluidics-based massively parallel snRNA-seq method, free of enzymatic dissociation and nucleus sorting
**spatial single-cell omics**	mRNA, protein	spatiotemporal molecular and dynamic transcriptomic changes
**seq-FISH**	mRNA, protein	different fluorescent probes to characterize spatial organization of cells
**scATAC-seq**	chromatin	tag and fragment DNA sequences in open chromatin regions with DNA transposase (Tn5)
**scDNase-seq**	chromatin	DNase I that digest chromatin fragmentation
**scMNase-seq**	chromatin	MNase for detecting chromatin accessibility and nucleosome position
**iscDNase-seq**	DNA	barcoding DNA ends with TdT terminal transferase and T4 DNA ligase combined
**CITE-seq**	mRNA, protein	antibody conjugates bound to biotinylated DNA barcodes

Table 2. Summary of the main characteristics of the technologies described in the review.
